# Formation and Application of Core–Shell of FePt-Au Magnetic–Plasmonic Nanoparticles

**DOI:** 10.3389/fchem.2021.653718

**Published:** 2021-04-27

**Authors:** Da-Hua Wei, Tei-Kai Lin, Yuan-Chang Liang, Huang-Wei Chang

**Affiliations:** ^1^Department of Mechanical Engineering, Institute of Manufacturing Technology, National Taipei University of Technology (TAIPEI TECH), Taipei, Taiwan; ^2^Department of Optoelectronics and Materials Technology, National Taiwan Ocean University, Keelung, Taiwan; ^3^Department of Physics, National Chung Cheng University, Chiayi, Taiwan

**Keywords:** FePt-Au nanoparticles, surface modification, thiol, magnetic-plasmonic, magnetic heating hyperthermia

## Abstract

Monodispersed FePt core and FePt–Au core–shell nanoparticles (NPs) have been chemically synthesized in liquid solution and with controllable surface-functional properties. The NP size was increased from 2.5 nm for FePt to 6.5 nm for FePt–Au, which could be tuned by the initial concentration of gold acetate coated onto FePt seeding NPs via a seed-mediated formation of self-assembled core–shell nanostructures. The analyses of the interplanar spacing obtained from the high-resolution transmission electron microscopy (HRTEM), selective electron diffraction pattern (SAED), and x-ray diffraction (XRD) confirmed that both FePt core and Au shell belong to the face-centered cubic (fcc) structure. FePt–Au NPs have a surface plasmon resonance (SPR) peak at 528 nm in the visible optical band region, indicating the red shift compared with the typical theoretical value of 520 nm of pure Au NPs. The surface modification and ligand exchange of FePt–Au was using mercaptoacetic acid (thiol) as a phase transfer reagent that turned the NPs hydrophilic due to the functional carboxyl group bond on the surface of presented multifunctional magnetic–plasmonic NPs. The water-dispersible FePt-based NPs conjugated with biomolecules could reach the different biocompatibility requirements and also provide enough heating response that acted as a potential agent for magnetic fluid hyperthermia in biomedical engineering research fields.

## Introduction

Hybrid metal nanoparticles (NPs) with a magnetic core and multifunctional shell have been explored extensively owing to their wide-range research applications such as information storage and other biological functions in marker, separation, labeling material, drug delivery, therapy, and biological targeting (Lyon et al., 2004; Caruntu et al., 2005; Piao et al., 2008; Kim et al., 2010; Bao et al., 2016; Kudr et al., 2017; Yang et al., 2018; Chai et al., 2019). Compared with non-magnetic NPs, magnetic NPs provide a characteristic of flexible constitution on controlling and manipulation by an external magnetic field to satisfy more biomedical assays. Recently, chemically synthesized iron-based NPs and related core–shell NPs in organic solvents have been extensively studied including fundamental and functional interests due to their attractive optical, electronic, photocatalytic, biological, energy-saving, magnetic resonance imaging (MRI), and magnetic–plasmonic applications (Yavuz et al., 2006; Gao et al., 2007; Zeng and Sun, 2008; Levin et al., 2009; Chou et al., 2010; Wei and Yao, 2011; de la Presa et al., 2012; Chen et al., 2013; Seemann and Kuhn, 2014; Zhuang et al., 2015; Mandal and Chaudhuri, 2016; Shu et al., 2017; Nemati et al., 2018; You and Guo, 2019; Chan et al., 2020). So the simple and reproducible methods to control the crystallite size, composition, and related nanoshell coatings over the magnetic core with tunable plasmonic surface characteristics of the core–shell NPs are very important due to the fact that all potential developments are directly dependent on such corresponding magnetic–plasmonic character statements (Xu et al., 2007; Li et al., 2020a). On the other hand, the synthesis of magnetic–plasmonic hybrid nanostructures via coupling magnetic and plasmonic anisotropy could provide an advanced approach for designing multifunctional devices with the desired selective plasmonic excitation by magnetic fields (Li et al., 2020b). The advantage of the solution-phase synthesis is control over the particle size and its distribution close to atomic scale and atomic precision, which strongly affects the chemical stability and biocompatibility of the synthesized NPs. Magnetic iron–platinum (FePt) NPs have suitable magnetocaloric ability to provide enough heating energy and thus destroy cancer cells for potential applications in chemotherapy and magnetic fluid hyperthermia (MFH) (Chou et al., 2010; Chan et al., 2020).

On the other hand, gold nanostructures were widely used to exhibit surface plasmon resonance (SPR) and good biocompatibility, causing optical extinction at a visible wavelength (Durr et al., 2007; Chen et al., 2010). Therefore, Au-coated magnetic composite core–shell NPs have attracted more considerable attention and interest due to enhanced chemical stability in biomedical development avoiding oxidation and corrosion from the core, which could also exhibit unique optical and magnetic properties and satisfy water compatibility via surface modification. A general seed-mediated method to the synthesis of hydrophilic and biocompatible Au–Fe_3_O_4_ heterodimers was reported (Zeng et al., 2019), in which the size of metals and Fe_3_O_4_ can be independently regulated in a wide range due to the fact that magnetic/plasmonic hybrid nanoparticles are highly desirable for multimodal bioimaging and biosensing. The use of Au–Fe oxide NPs as heat generators was reported to combine with magnetic hyperthermia (MHT) and photothermal therapy (PTT) protocols, according to varied parameters such as magnetic and plasmonic NP design, NP concentration, and exposure settings, which have emerged as a promising biomedical manipulation (Espinosa et al., 2020). Herein, we propose the magnetic–plasmonic FePt–Au core–shell NPs that can be used as a potential heating agent for applications such as cancerous or tumor hyperthermia (Chen et al., 2013; Chan et al., 2020). Therefore, the Au shell could provide several advantages as a functional coating on the core surface due to its low chemical reactivity and excellent ability to form self-assembled monolayers (SAMs) on their surface structure by using organic alkanethiol radicals, meaning long-chain thiols on gold (Sun et al., 2008).

Uniformity in size and shape of inorganic NPs is usually synthesized under a hydrophobic state using hydrocarbon or fluorocarbon chains, causing the NPs to be immiscible in aqueous solutions. In order to satisfy the biological applications, it is required to develop methods to transfer the organic magnetic NPs into aqueous solutions that could be easily dispersed in blood to exhibit good biocompatibility and be directed to a specific target toward applying an external magnetic field. In this present work, FePt–Au core–shell nanocomposites with organic ligands were firstly synthesized in order to understand the Au shell effect on the fundamental properties of the FePt core. Then, FePt–Au NPs were phase transferred into aqueous solutions via a ligand-exchange procedure to satisfy the request of biocompatibility, and they could be easily controlled by an external field to produce local heat as a potential heating agent for magnetically induced hyperthermia applications. The method of heat hyperthermia has been accepted as a potential therapeutic modality for treating malignant tumors such as destroying cancer cells, not with traditional drugs, outside the human body.

## Synthesized Procedures of FePt-Au Nanoparticles

All the reagents used in synthesis were commercial sources and without further purification, which were purchased from Sigma Aldrich. The following are for the FePt NPs using airless synthesis with the reduction: 0.5 mmol for platinum acetylacetonate Pt(acac)_2_, 0.75 mmol for iron acetylacetonate Fe(acac)_3_, and 3.75 mmol 1,2-hexadecanediol were mixed with 30 ml of phenyl ether. After purging with argon for 0.5 h at room temperature, the flask was heated up to 100°C for 0.5 h with additive oleic acid (OA, 0.75 mmol) and oleylamine (OL, 0.5 mmol) stabilizers into the flask at the same time (Wei et al., 2017; Lin et al., 2018; Chan et al., 2020). Then, the mixture was heated up to 260°C for 1 h to form FePt NPs. The mole ratio of 1 mmol FePt NPs was then used as a seeding material for the reduction of gold acetate Au(ac)_3_ (0.2~0.8 mmol) to form gold-coated FePt NPs denoted as core–shell of FePt–Au (0.2~0.8) NPs. The Au-mixed FePt seeding solution was reacted at 260°C for 1 h, and the final composite solution appeared to be dark purple. The surface modification of the FePt-Au NPs transferred from oil- to water-soluble state was prepared with mercaptoacetic acid (thiol, C_2_H_4_O_2_S) simulated in human body fluid and confirmed by Fourier transform infrared spectroscopy (FTIR) spectral analysis. The crystalline structure and particle sizes were identified by *ex situ* x-ray diffraction (XRD) and transmission electron microscopy (TEM), respectively. The magnetic properties were characterized at room temperature using the vibrating sample magnetometer (VSM) with the applied field up to 20 kOe. The absorption spectra for all NP dispersions were measured using the UV-vis spectrometer.

## Results and Discussion

**Figure 1:**
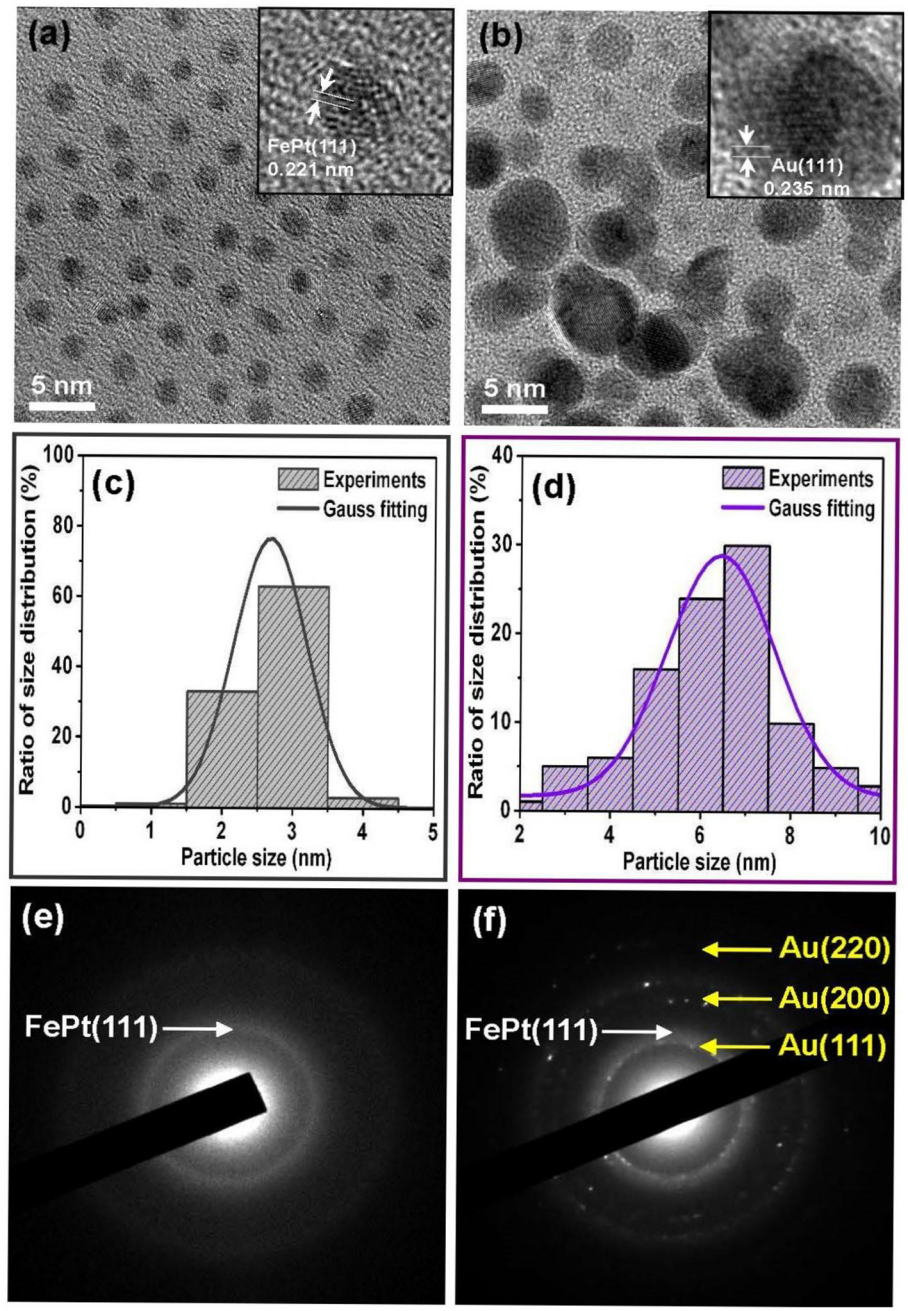
Representative TEM bright-field images are for the as-synthesized **(a)** FePt and **(b)** FePt–Au core–shell NPs, and the inset TEM images of corresponding high-resolution transmission electron microscopy (HRTEM) of individual NPs, respectively. **(c,d)** are the corresponding range of histograms with the Gauss fitting curve for evaluating the average particle size and its distribution, respectively. **(e,f)** are the typical corresponding selective electron diffraction patterns.

Figure 1 shows the TEM bright-field images for as-synthesized (a) FePt and (b) FePt–Au(0.2) core–shell NPs, and the inset TEM images are the corresponding high-resolution transmission electron microscopy (HRTEM) micrographs of individual FePt core and FePt–Au core–shell NPs, respectively. (c,d) are the corresponding range of histograms with the Gauss fitting curve for evaluating the average particle diameter and its distribution, respectively. (e,f) are the corresponding selective electron diffraction patterns (SAED). The FePt NPs capped with oleic acid and oleylamine ligands could be monodispersed in hydrophobic solvents without significant aggregation, as observed in Figure 1a. The average diameter of the as-synthesized FePt core NPs was about 2.5 nm with a narrow size distribution shown in Figure 1c, and the particles in fraction Figure 1a range in size from 2 to 3 nm, with an average size of 2.5 ± 0.5 nm as shown in Figure 1c. For the FePt NPs after coating with 0.2 mmol Au, the FePt–Au NPs became much darker than pure FePt core NPs and started to aggregate and form coalesced-like NPs, as shown in Figure 1b. It can also be seen that the particle contrast has been changed with the increase in particle size. The average diameter of NPs changed from 2.5 nm for pure FePt core NPs to about 6.5 nm for FePt–Au core–shell NPs as shown in Figure 1d, and the NPs in fraction Figure 1b contain NPs ranging in size from 4 to 9 nm, with an average particle size of 6.5 ± 2.5 nm as shown in Figure 1d. The interplanar spacing of 0.221 nm obtained from the HRTEM image can be ascribed to the adjacent (111) plane of the FePt disordered crystal. On the other hand, the interplanar spacing of 0.235 nm obtained from the HRTEM image can be ascribed to the adjacent (111) plane of the face-centered cubic (fcc) structure belonging to the Au shell lattice type. The SAED diffraction analysis for FePt NPs is with the (111) plane in the face-centered cubic (fcc) FePt as shown in Figure 1e. The coexistence of FePt NPs and crystalline Au is shown in Figure 1f. The SAED pattern of a group of particles shows both the fcc Au and fcc FePt diffraction rings in the selected area, suggesting the existence of FePt–Au core–shell NPs. According to the feature contrast, the increase in size and slight change in shape of the FePt NPs coated with Au in the TEM and HRTEM images provide the clear evidence that supports the formation of FePt–Au core–shell nanostructured materials based on this present in the Au concentration-dependent self-assembly process.

**Figure 2:**
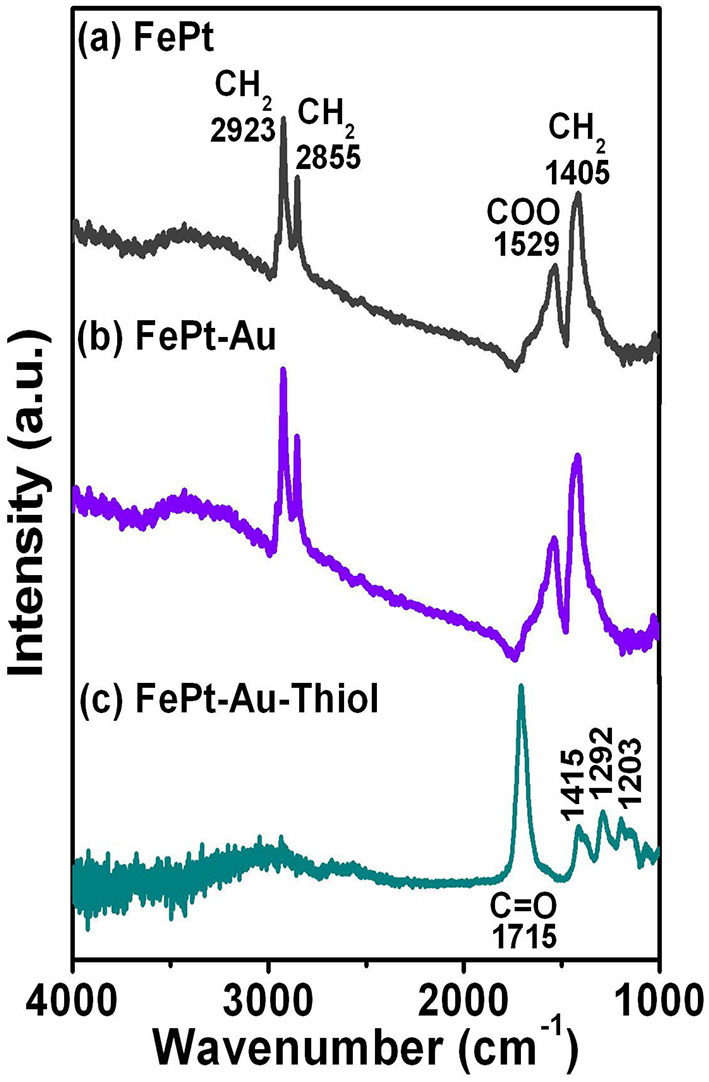
FTIR spectra for the **(a)** FePt and **(b)** FePt-Au(0.2) NPs synthesized in phenyl ether capped with oleic acid and oleylamine, respectively. **(c)** FePt-Au(0.2) NPs were ligand exchanged with thiol for water solubility.

Figure 2 shows Fourier transform infrared spectroscopy (FTIR) spectra for (a) FePt and (b) FePt-Au(0.2) NPs synthesized in phenyl ether capped with oleic acid (OA) and oleylamine (OL), and then the (c) FePt-Au(0.2) NPs were ligand exchanged with thiol. The spectra of FePt and FePt-Au NPs reveal modes characteristic of the oleyl group: the peaks at 2855 and 2923 cm^−1^ are due to the symmetric and asymmetric CH_2_ stretching modes of aliphatic chains. The appearance of both peaks at 1529 and 1405 cm^−1^ is due to the vibrational ν(COO) and stretching (CH_2_) modes, respectively. The above results indicated that OA and OL complex surfactants bonded to the as-synthesized FePt and FePt–Au NPs, thus causing the presence of bidentate ligands bonding to the as-synthesized NPs shown in Figures 2a,b. For the FePt–Au NPs ligand exchanged with thiol, the strong peak at 1715 cm^−1^ was observed due to the C=O stretch vibration mode of alkyl thiol chains as well as weaker absorption peaks at 1415, 1292, and 1203 cm^−1^ as shown in Figure 2c. The disappearance peaks of CH_2_ stretching modes indicate the changes in the surface chemistry of the FePt-Au NPs (Kikuchi et al., 2011). The FTIR spectra for FePt–Au NPs after ligand exchange with thiol are indeed different compared to the as-synthesized ones, which confirms the oleic acid and oleylamine ligands replaced by carboxyl groups attributed to the thiol treatment as shown in Figures 2b,c. The reagent containing the thiol functional group was facilely approached to replace the surfactant on the definite metal surface which also confirms that thiol bonds to the surface of the FePt–Au NPs, making the FePt-based NPs stable in water solution. This kind of thiol functional groups at the surface could enhance NP solubilization in various solvents, extending their potential applications in many fields. It is well-known that the Au surface can be easily functionalized with thiol groups. This method allows the linkage of functional ligands which may make the multifunctional materials suitable for sensing, detection, and biomedicine fields. This ligand-exchange procedure for the functionalization of composite NP surface is for tuning the overall properties of particles to fit targeted, catalytic, and bio-optical applications.

**Figure 3:**
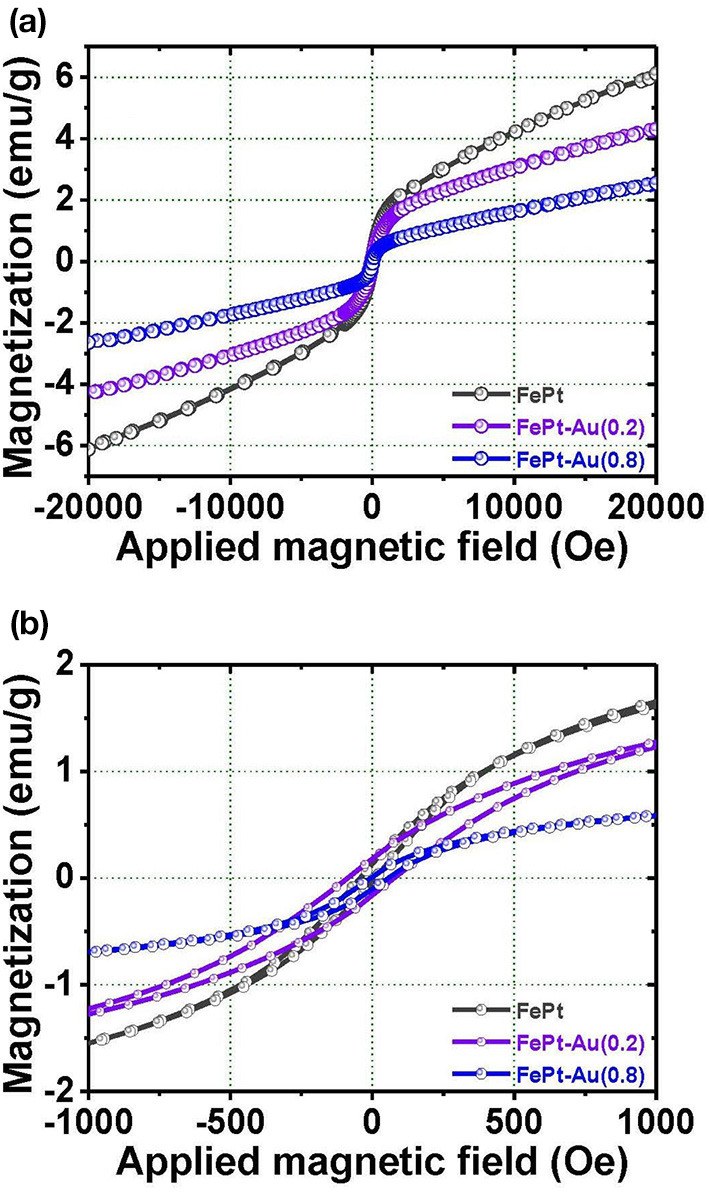
The magnetization loops for the **(a)** FePt, FePt-Au(0.2), and FePt-Au(0.8) NPs measured at room temperature, respectively. **(b)** The inset shows the corresponding enlarged hysteresis loops for FePt, FePt-Au(0.2), and FePt-Au(0.8) NPs ranging ±1000 Oe in the field, respectively.

Figure 3a shows VSM hysteresis loops of the FePt, FePt-Au(0.2), and FePt-Au(0.8) NPs measured at room temperature, respectively. Figure 3b shows the corresponding enlarged hysteresis loops for FePt, FePt-Au(0.2), and FePt-Au(0.8) NPs ranging ±1000 Oe in the field. The FePt NPs without and with Au coating are at superparamagnetic in nature. The magnetization values are 6.1 and 4.3 emu/g for FePt and FePt-Au(0.2) NPs, and then decreased to 2.5 emu/g for FePt-Au(0.8) NPs, respectively. The magnetization value is decreased with the increase of the Au concentration-dependent FePt-Au NPs. In order to obtain a better heating capability of FePt–Au NPs for magnetic fluid hyperthermia application, FePt-Au(0.2) has been selected for further investigation due to the fact that the heating efficiency is directly proportional to the magnetization value related to the strong dependence of the magnetic nanoparticle concentration in the core–shell nanostructure (Chan et al., 2020; Espinosa et al., 2020). So the following analyses will be focused on the FePt structures without and with Au(0.2) over FePt in order to study the effects of Au coating on the magnetic and optical performance of the FePt-based NPs. The magnitude of the magnetization for FePt–Au is much smaller than that of FePt NPs, and the magnetization values of FePt–Au at all fields are lower than those of pure FePt NPs. It can be understood that Au is a non-magnetic element, and it dilutes the total magnetization of FePt–Au NPs. This variation of magnetization value is believed to reflect the decreased coupling of the magnetic moments as a result of the increased interparticle spacing of magnetic cores, which is due to a combination of the gold and the organic capping shells for FePt–Au. The similar concept and results were also reported by M–Fe_3_O_4_ (M = Au and Ag) core–shell heterodimer NPs (Wang et al., 2005; Jiang et al., 2008). From the enlarged hysteresis loops, another important difference in coercivities between FePt and FePt-Au NPs can be identified. The coercivity values are 28 and 86 Oe for FePt and FePt–Au(0.2) NPs, respectively. The coercivity showed a clear increase for FePt–Au(0.2) NPs. This observation likely reflects the fact that coercivity of a superparamagnetic NP is related to the particle size (Boal et al., 2004). Therefore, the increase in the coercivity can be attributed to the larger size of Au-coated FePt that leads to a less-effective coupling of the magnetic dipole moments. In contrast, the magnetic dipole moments in the pure FePt NPs are coupled more effectively, and hence the particles tend to have a lower coercivity.

**Figure 4:**
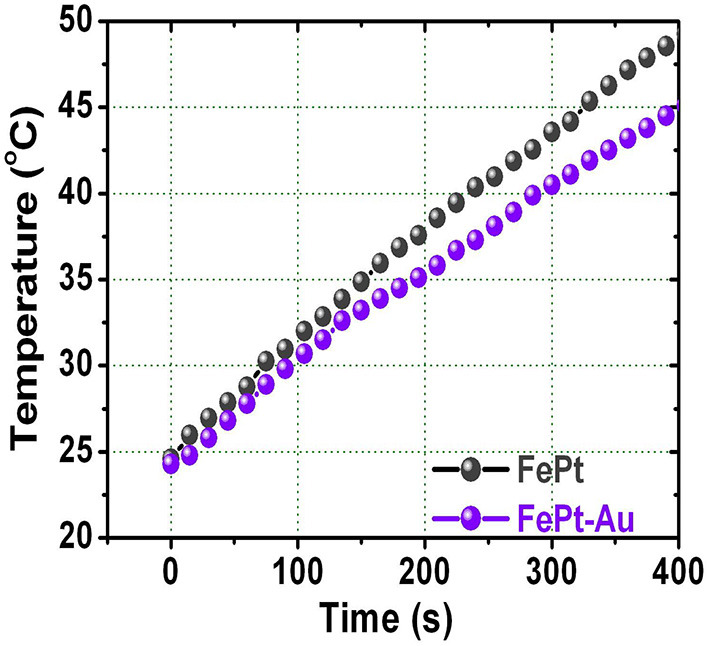
The relationship between raised temperature and the heating time for FePt and FePt-Au NPs dispersed in water solution, respectively.

Figure 4 shows the temperature response of the FePt core and FePt–Au core–shell NPs as a function of the heating time dispersed in water. The hydrophilic FePt and FePt–Au(0.2) NPs for temperature response were measured at 700 kHz of frequency and a 3.8 kAm^−1^ alternating current (AC) magnetic field, and all NPs are with a concentration of 1 mg·mL^−1^. The slightly lower heating response of FePt–Au compared with FePt NPs is due to its relatively lower magnetization to generate resonance losses. By decreasing the magnetization of the FePt–Au NPs, the conductivity decreases as a natural consequence of the decreasing volume fraction of elemental Fe. Hence, the heating rate of the FePt–Au system is directly related with the magnetic character. Both the hydrophilic FePt and FePt–Au magnetic NPs could provide enough local heat (at least 43°C) <380 s to kill cancerous cells for potential cancer therapy treatments (Alvarez-Berríos et al., 2014; Chan et al., 2020; Espinosa et al., 2020). The cancer cells are destroyed at temperatures higher than 43°C, whereas the normal cells can be kept at higher temperatures. These presented hydrophilic FePt–Au NPs with good biocompatibility could be acted as a potential heating agent for hyperthermia or carrier in biological applications. So the major technical difficulty associated with heat-based cancer therapy is to heat only the tumor tissues without damaging the healthy ones in future work.

**Figure 5:**
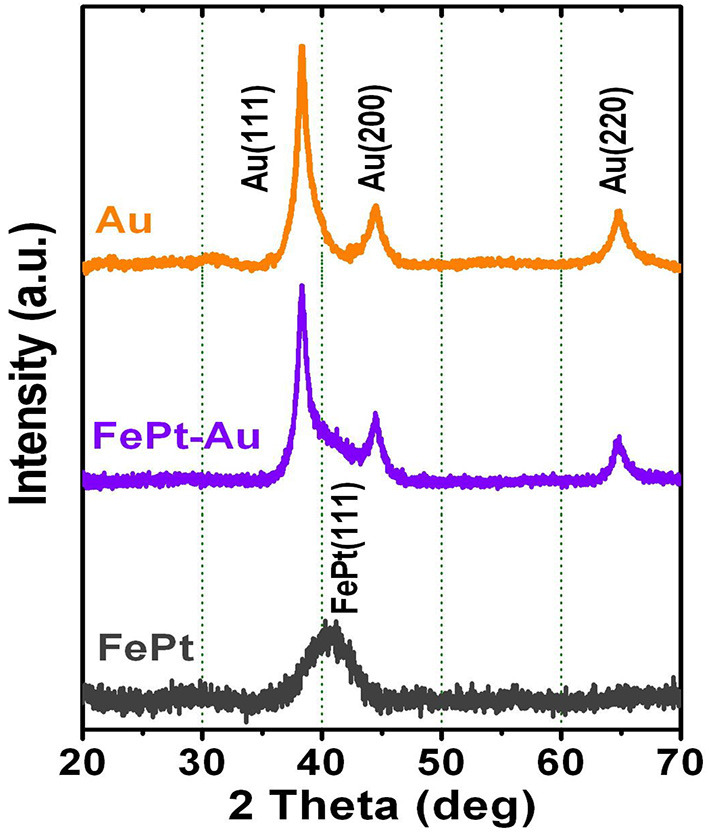
X-ray diffraction patterns for the FePt, FePt-Au, and Au NPs, respectively.

Figure 5 shows XRD patterns for the FePt, FePt–Au, and pure Au NPs, respectively. The as-synthesized FePt seeding NPs are without any external energy to overcome the activation energy of an ordered phase transformation, indicating the formation of the chemically disordered fcc FePt structure with (111) orientation. The FePt (111) diffraction peak for the pure FePt is relatively broad with a wider width compared to FePt–Au NPs, indicating that FePt NPs have a finer particle size than FePt–Au NPs. The XRD patterns for pure Au NPs have (111), (200), and (220) Au diffraction peaks of typical fcc Au crystals. The shoulder between Au(111) and Au(200) peaks for the FePt–Au NPs is due to the heavy atom effect from the Au shell that supports the formation of Au-coated FePt NPs, which can be indexed to FePt–Au core–shell nanostructures in a cubic phase. On the other hand, no diffraction peak of unclear phase or peak shift of FePt and Au from FePt–Au core–shell NPs was observed, thus providing strong evidence for complete coverage of the FePt core by Au shell without an intermixed phase. The above structural and morphological result analyses support the configuration of FePt–Au core–shell NPs, and the XRD pattern analysis consisted with the observation from the TEM images as shown in Figure 1.

**Figure 6:**
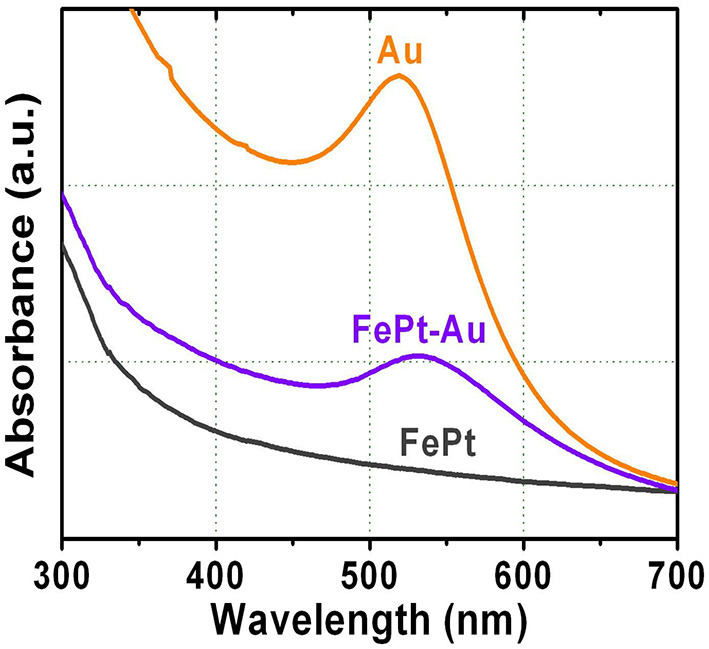
UV-vis spectra of the surface plasmon resonance band for the FePt, FePt-Au, and Au NPs dispersed in water solution, respectively.

The other important evidence for supporting the configuration of FePt–Au core–shell NPs is provided by the UV-vis spectra of the surface plasmon resonance (SPR) band compared with the pure FePt and Au NPs, respectively. The influence of the Au shell on the intensity of the gold plasmon peak was caused by the collective oscillations of free electrons, called surface plasmon, which induced an absorption peak to appear in the visible region of the electromagnetic spectrum. Figure 6 shows the SPR spectra for the FePt, FePt–Au, and Au NPs dispersed in water solution, respectively. No SPR band in the visible region from FePt NPs can be seen. The Au NPs have a strong absorption band in the visible region at about 520 nm. The FePt–Au NPs show a clear SPR band (528 nm) in the visible region due to the characteristic of the unique optical property from Au coating. The SPR band of FePt–Au NPs has shown a higher wavelength (red shift) in comparison with the pure Au NPs, indicating that the surface absorption varied after Au coating which also supports the configuration of FePt–Au core–shell NPs. This phenomenon suggests that there is unlikely a significant amount of pure Au or other mixed NPs in the presented FePt–Au core–shell NPs, while similar wavelength shifts have been reported for gold-coated iron oxide NPs prepared in aqueous solution (Qiu et al., 2009; Chen et al., 2018). The SPR plays a key role in the optical characteristics of metals, indicating that the localized heating effect could be generated from the illumination of the Au shell with infrared light, which can also be used in the field of photothermal therapy (PTT) (Chen et al., 2013; Cooper et al., 2014; Espinosa et al., 2020). On the other hand, the great potential of trimetallic FePt–Au NPs as highly efficient catalysts for promoting formic acid oxidation reaction (FAOR) of organic molecules was reported, providing a general approach to advanced NP catalysts with simultaneous enhancement in both activity and durability for practical applications (Zhang et al., 2012). So these photothermal phenomena and enhanced electro-oxidation reactions by the Au element could be a potentially encouraging application from our presented FePt–Au heterostructured core–shell NPs in the near future.

## Conclusions

The chemically synthesized core–shell of FePt–Au NPs with monodispersity by using the seed-mediated method is presented in this work, and all material analyses confirmed the formation of the FePt–Au core–shell nanostructure. The water-soluble FePt–Au NPs are generally considered to be biocompatible via ligand exchange by thiol, which could be potentially used for multiple applications. The hydrophilic FePt–Au NPs that bonded with carboxyl groups can be conjugated with various functional groups using linkers such as functional antibodies to target specific cells or tissues in aqueous solution as simulated in human body blood. On the other hand, the heating response of hydrophilic FePt–Au NPs could provide enough local heat to kill cancerous cells for potential cancer therapy treatment. We think that such a combination of optical and magnetic properties in FePt–Au magnetic–plasmonic material would enable simultaneous biolabeling/imaging and cell sorting/separation including corresponding magnetic, optical, catalytic, and biological fields in the future.

## Data Availability Statement

The original contributions presented in the study are included in the article/Supplementary Material, further inquiries can be directed to the corresponding authors.

## Author Contributions

D-HW: conceptualization, methodology, data curation, writing-review and editing, funding acquisition, and supervision. T-KL: methodology. Y-CL: funding acquisition and resources. H-WC: resources. All authors contributed to the article and approved the submitted version.

## Conflict of Interest

The authors declare that the research was conducted in the absence of any commercial or financial relationships that could be construed as a potential conflict of interest.
